# A Case of Intermittent Organo-Axial Gastric Volvulus

**DOI:** 10.7759/cureus.9387

**Published:** 2020-07-25

**Authors:** Dhineshreddy Gurala, Fady G. Haddad, Liliane Deeb

**Affiliations:** 1 Internal Medicine, Staten Island University Hospital, Northwell Health, Staten Island, USA; 2 Gastroenterology and Hepatology, Staten Island University Hospital, Staten Island, USA

**Keywords:** stomach, malrotation, obstruction

## Abstract

Gastric volvulus is defined as twisting of the stomach along its long or short axis, a rare clinical entity that can lead to gastric outlet obstruction. Symptoms are typically acute and include retching, abdominal pain, and vomiting. Chronic intermittent manifestations of this entity present a diagnostic challenge as conclusive imaging findings are only apparent during symptomatic periods. Given the disorder’s intermittent nature, a volvulus may resolve before imaging studies can be conducted. We present a rare case of an intermittent organo-axial gastric volvulus that responded to conservative measures.

## Introduction

Gastric volvulus, which can be acute or chronic, is described as torsion of the stomach leading to partial or complete closed-loop obstruction. Acute gastric volvulus presents in 70% of cases with a triad (known as Borchardt’s triad) of symptoms, including retching without emesis, abdominal pain, and inability to pass a nasogastric tube [1]. It is a rare surgical emergency, and attributed mortality could range from 30% to 50% due to missed diagnosis and delayed treatment [2]. The incidence of gastric volvulus peaks after the fifth decade, with adults constituting 80% to 90% of cases [3]. It is subclassified based on the axis of rotation into organo-axial, mesentero-axial, or combined. Organo-axial malrotation constitutes 59% of cases of gastric volvulus [4]. Acute organo-axial malrotation of greater than 180 degrees leads to complete obstruction, whereas a lesser degree of rotation results in partial obstruction. Chronic intermittent organo-axial malrotation is difficult to diagnose due to the unpredictable nature of the disorder. Surgical and endoscopic de-rotation are the primary modalities of treatment. We present a case of an intermittent organo-axial gastric volvulus that responded to conservative measures. This case was presented as an abstract at the 2019 ACG Annual Scientific Meeting Abstracts (Gurala D, Fady H, Deeb L. A Case of Intermittent Organo-Axial Gastric Volvulus. Program No. P2686. ACG 2019 Annual Scientific Meeting Abstracts. San Antonio, Texas: American College of Gastroenterology, October 29, 2019).

## Case presentation

A 92-year-old woman presented to the emergency department with chief concerns of intermittent epigastric pain and food intolerance lasting several weeks. She had a history of dysphagia one year before this admission, for which she underwent upper gastrointestinal (GI) series initially followed by an upper GI endoscopy. Endoscopy demonstrated the narrowing of gastric lumen without any twisting or signs of ischemia; however, the upper GI series showed a paraesophageal hiatal hernia and an organo-axial rotation of the stomach without obstruction. Her symptoms resolved at that time without intervention. Other past medical history was significant for atrial fibrillation. Her vital signs were stable. A physical examination revealed cachexia and epigastric tenderness without guarding, rigidity, or rebound tenderness. Her laboratory test results were within reference ranges.

**Figure 1:**
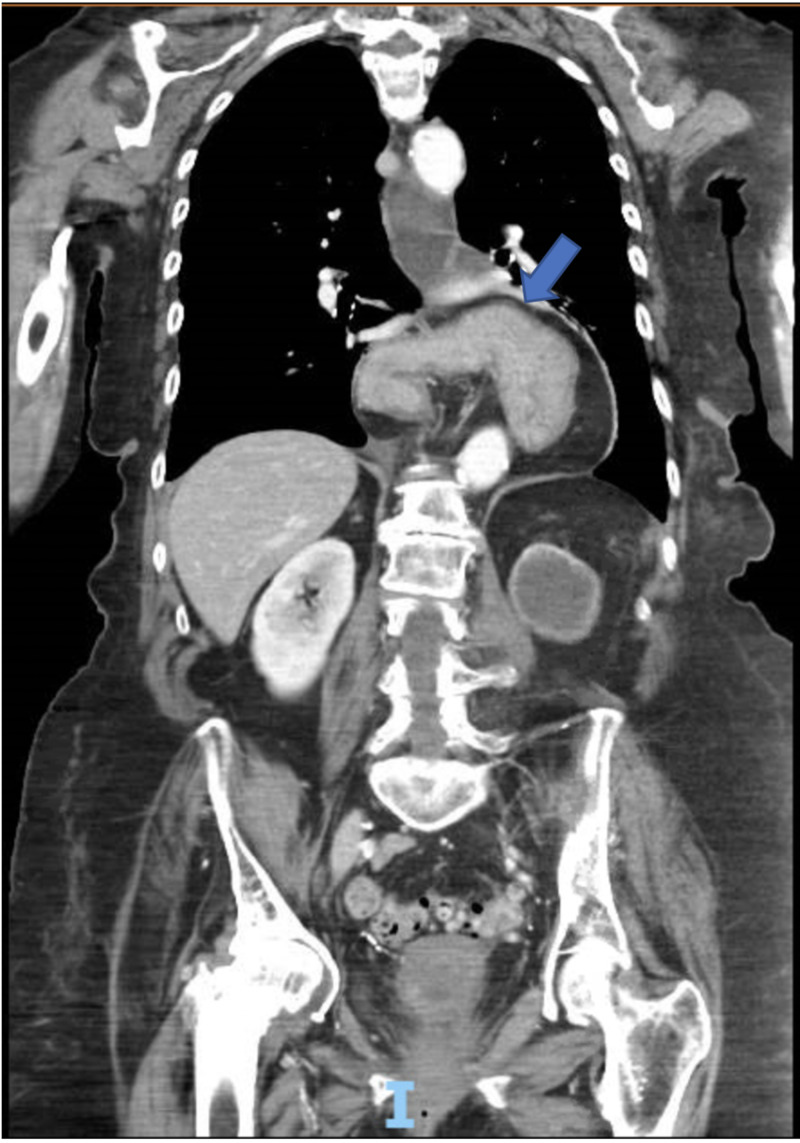
CT of the abdomen (axial view) showing organo-axial malrotation of the stomach.

A plain radiograph of the abdomen showed a non-obstructive bowel gas pattern without evidence of pneumoperitoneum. A contrast-enhanced CT scan of the abdomen and pelvis revealed a large hiatal hernia with an intrathoracic stomach and organo-axial malrotation (Figure 1).

**Figure 2:**
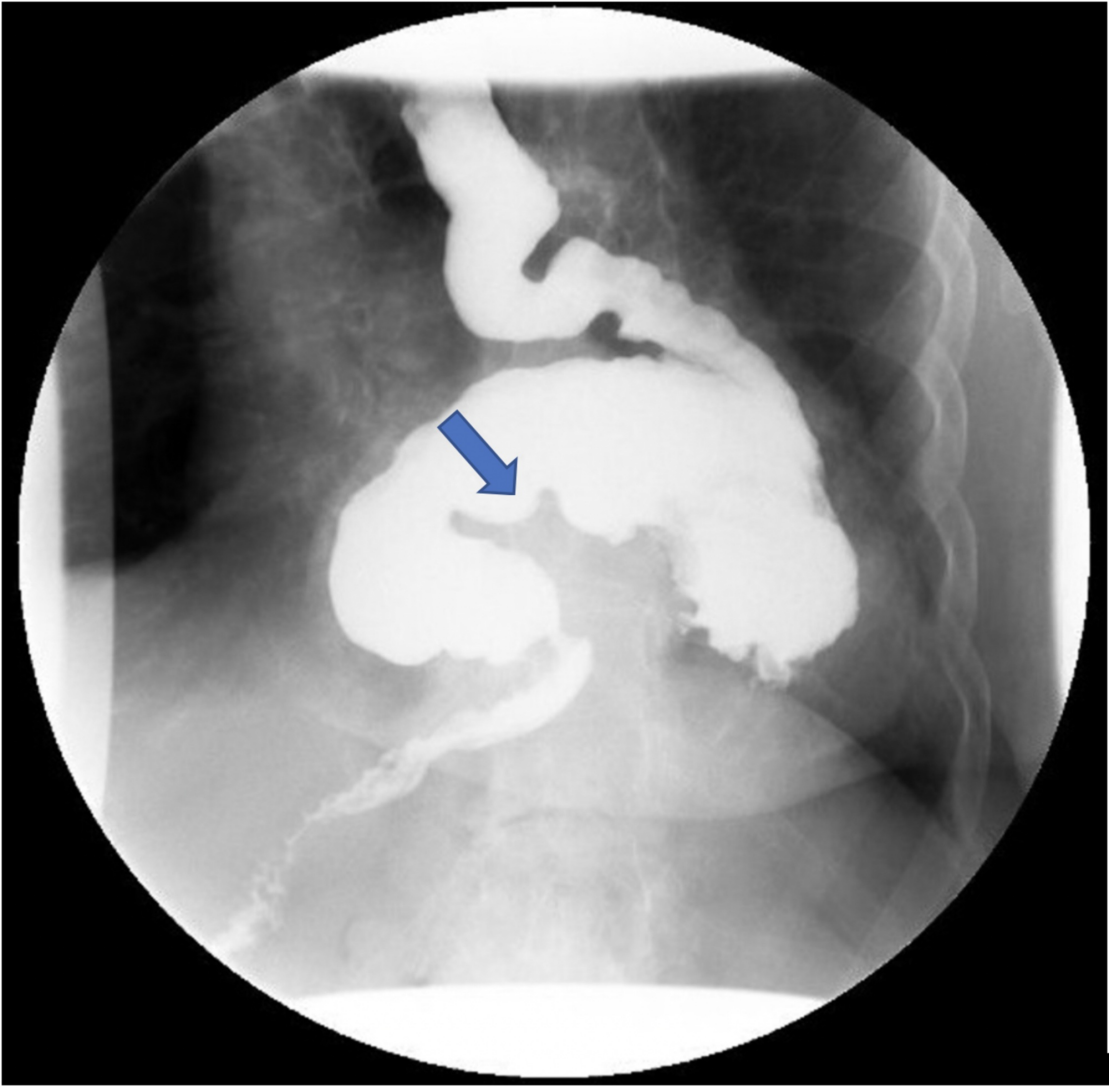
Upper GI series using barium showing organo-axial malrotation of the stomach.

**Figure 3:**
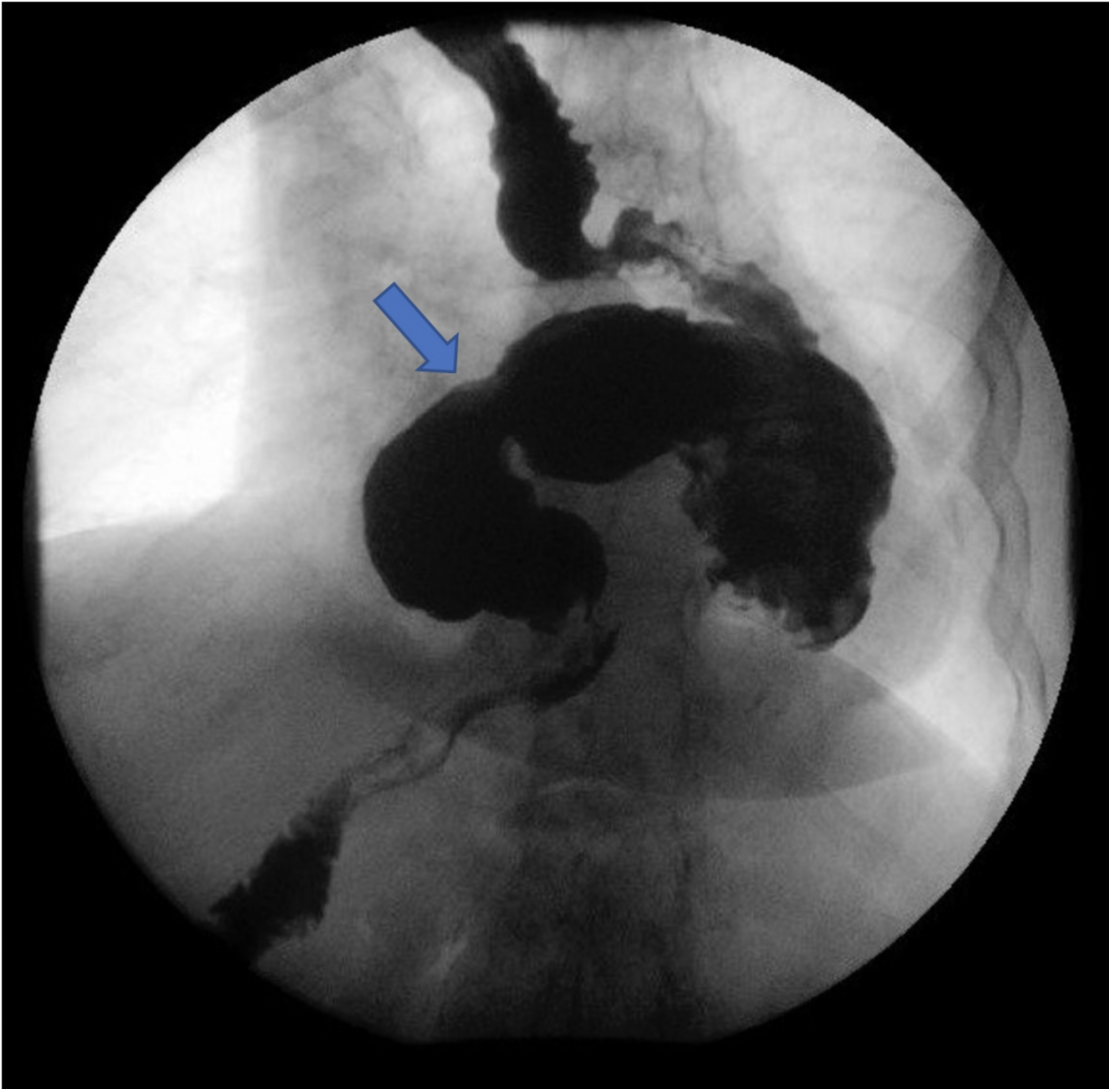
Upper GI series using gas showing organo-axial malrotation of the stomach.

Upper GI series showed a large paraesophageal hernia with an organo-axial rotation of the stomach with partial obstruction at the gastroduodenal junction level (Figures 2, 3).

The patient was started on intravenous hydration and nothing by mouth (i.e., nil per os). General surgery consultants recommended surgical (gastropexy) or endoscopic intervention; however, the family refused both treatment options given her poor functional status. Her symptoms resolved progressively on conservative measures, and the patient was able to tolerate an oral diet within two to three days. She was subsequently discharged to a specialized nursing facility five days after admission.

## Discussion

According to Milne et al., gastric volvulus was first described by Berit in 1866 as a torsion of the stomach around its short or long axis [3]. Based on the axis of rotation, Singleton subclassified the condition into organo-axial, mesentero-axial, or combined volvulus [3]. Organo-axial volvulus results from rotation along the long axis from the gastroesophageal junction to the pylorus. Mesentero-axial rotation is characterized by rotation along the transverse axis such that the pylorus rotates above the gastroesophageal junction. Combined volvulus results from a combination of both forms of rotation. Approximately 59% of volvulus cases are organo-axial, 29% are mesentero-axial, and 12% are combined [5]. Mesentero-axial volvulus occurs more commonly in the pediatric population [4]. Organo-axial volvulus is more common in adults, with the peak incidence occurring after the fifth decade. It is frequently seen in the context of diaphragmatic abnormalities such as paraesophageal hernia, hiatal hernia, or diaphragmatic eventration.

The clinical presentation of gastric volvulus depends on the onset and degree of obstruction. A rotation greater than 180 degrees results in complete obstruction, whereas rotation less than 180 degrees results in partial obstruction. Upper abdominal distension with dullness to percussion is seen with complete gastric outlet obstruction (GOO) [6,7]. In some cases, the acute presentation of volvulus may be confused with acute coronary syndrome, which can result in delayed diagnosis [8,9]. Chronic gastric volvulus presents with vague symptoms that spontaneously resolve, including upper abdominal distension, early satiety, gastroesophageal reflux, and intermittent dysphagia. These intermittent symptoms may be confused with peptic ulcer disease or cholecystitis. Diagnosis is often difficult and delayed due to the intermittent nature of this disorder, as well as the fact that abnormalities on imaging are present only during symptomatic periods. Heightened awareness is warranted in such cases to avoid delayed management and subsequent complications.

For a suspected diagnosis based on the history and physical examination, an abdominal film is the initial test that can confirm the presence of a sizable spherical bubble in the upper abdomen or chest with air-fluid levels [10]. If the x-ray of the abdomen is not revealing, a CT should be the next imaging of choice. If the diagnosis is still in question, an upper GI barium test is recommended.

Acute gastric volvulus is a surgical emergency with a mortality rate between 30% and 50% if the diagnosis is missed and management is delayed [11,12]. Complications associated with volvulus include bowel obstruction, strangulation, ischemia, necrosis, and perforation. Strangulation is the most common complication, occurring in 30% of cases [12]. Initial management consists of stabilizing the patient (via fluid resuscitation and correction of electrolytes), bowel rest, and nasogastric tube placement to reduce distension. Primary surgical management involves decompression of the stomach, volvulus reduction, and possible gastropexy or gastrostomy tube placement, in addition to the correction of any underlying intra-abdominal defects. In patients with multiple comorbidities and high surgical risk, the less invasive endoscopic approach is recommended. This intervention involves endoscopic de-rotation and gastric fixation with one or two gastrostomy tubes [13]. In the latter approach, however, repair of anatomical defects is lacking. 

In our patient with chronic intermittent organo-axial volvulus causing partial obstruction, surgical management with gastropexy along with the repair of her paraesophageal hernia or an endoscopic approach to accomplish de-rotation would be an appropriate choice of treatment. However, due to her age, multiple comorbidities, and poor functional status, the patient and her family declined to pursue any aggressive measures.

## Conclusions

Gastric volvulus is a rare clinical condition that can manifest either as an acute abdominal emergency or a chronic intermittent medical problem. Maintaining a high index of clinical suspicion is crucial due to the vague and intermittent presentation of this disorder, particularly in the absence of classic symptoms, as was the case with our patient. Heightened awareness is warranted when patients present with GOO in the setting of upper GI tract anatomical abnormalities, since delayed diagnosis and management may result in life-threatening complications and high mortality rates.
